# Atorvastatin associated with gamma glutamyl transpeptidase elevation in a hyperlipidemia patient: A case report and literature review

**DOI:** 10.1097/MD.0000000000022572

**Published:** 2020-10-02

**Authors:** Yan Xu, Yanqing Wu

**Affiliations:** Department of Cardiovascular Medicine, Institute of Cardiovascular Disease, Second Affiliated Hospital of Nanchang University, Nan Chang, Jiang Xi, 330006, PR China.

**Keywords:** γ-glutamyl transpeptidase (GGT), atorvastatin

## Abstract

**Rationale:**

Atorvastatin is the most common drug used in therapy for cardiovascular diseases. The most common adverse side effects associated with statins are myopathy and hypertransaminasemia. Here, we report a rare case of gamma glutamyl transpeptidase (GGT) elevation induced by atorvastatin.

**Patient concerns:**

A 47-year-old male was admitted to our hospital with dyslipidemia, he had been taking pitavastatin 2 mg/day for 2 months. The levels of total cholesterol (265.28 mg/dL) and low-density lipoprotein-cholesterol (LDL) (179.15 mg/dL) were also high.

**Diagnosis:**

Blood lipid test showed mixed dyslipidemia.

**Intervention:**

Atorvastatin 10 mg/day was given to the patient.

**Outcomes:**

The patient came back to our hospital for blood tests after 4 weeks. Although no symptoms were detectable, the patient's GGT level was markedly elevated (up to 6-fold over normal level) with less marked increases in alkaline phosphatase (ALP) and alanine aminotransferase (ALT). The serum GGT level returned to normal within 6 weeks of cessation of atorvastatin.

**Lessons:**

This is a case of GGT elevation without hyperbilirubinemia, hypertransaminasemiam, or serum creatine phosphokinase (CPK) abnormalities despite an atorvastatin regimen. This case highlights GGT elevation caused by atorvastatin, a rare but serious condition. Clinicians should be aware of these possible adverse effects and monitor liver function tests in patients on statin therapy.

## 1 Introduction

Statins are the most common drugs used for therapy for hyperlipidemia, coronary artery disease, and other atherosclerotic diseases in clinical practice.^[1]^ Statins have been shown to reduce cardiovascular events significantly in high-risk patients with and without hyperlipidemia.[^2^,^3^] However, despite the beneficial impact of the statins themselves, such adverse effects as myopathy, myalgia, cognitive impairment, and liver dysfunction have also been of great concern and have significant limitations that mitigate clinical applicability.[^4^,^5^] Atorvastatin (RLipitor) is a member of the medication class known as statins. Like all statins, atorvastatin works by inhibiting hydroxymethylglutaryl-coenzyme A reductase.

To the best of our knowledge, there have been no reported cases of atorvastatin-induced γ-glutamyl transpeptidase (GGT) elevation in the absence of hyperbilirubinemia, hypertransaminasemia, and serum creatine phosphokinase (CPK) abnormalities. We present here a case whose serum GGT level elevation was caused by atorvastatin.

## 2 Case presentation

**Figure 1:**
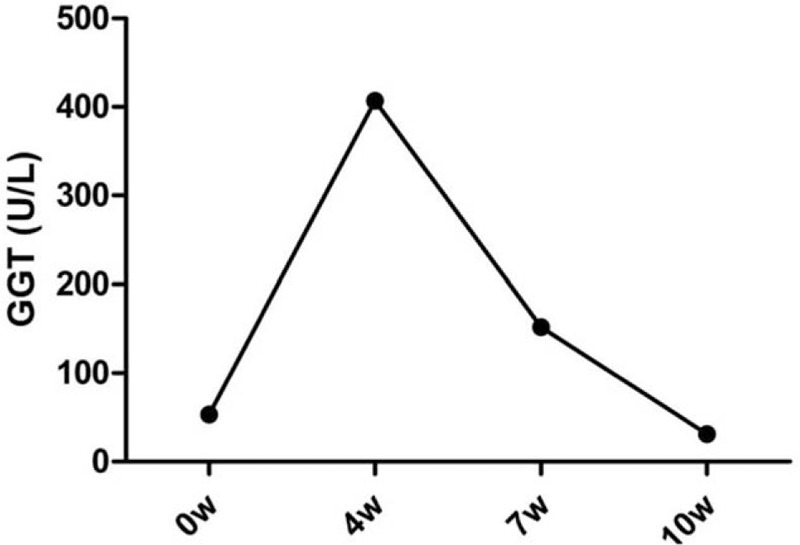
GGT levels starting from the week of admission.

A 47-year-old male was admitted to our hospital with dyslipidemia. He had been receiving pitavastatin 2 mg/day for 2 months. The levels of total cholesterol (265.28 mg/dL) and low-density lipoprotein-cholesterol (LDL) (179.15 mg/dL) were also high. The blood liver function test was normal. His height was 170.0 cm, and his weight was 65.0 kg, so his body mass index was 24.1. He had a history of smoking and no significant medical history of other conditions. He had no history of blood transfusions or alcohol intake. According to 2019 ESC/EAS Guidelines for the management of dyslipidemias, the patient had mid-level risk.^[6]^ Atorvastatin 10 mg/day was given to the patient, and he came back to our hospital for blood tests after 4 weeks. The result found his serum GGT level was markedly elevated (407 U/L, upper limit of normal: 61 U/L). The levels of serum alkaline phosphatase (ALP) (178 IU/L, upper limit of normal: 129 IU/L) and alanine aminotransferase (ALT) (51 U/L, upper limit of normal: 41 U/L) were slightly elevated, but the levels of serum CPK, total bilirubin (TBIL), and glutamic oxaloacetic transaminase (AST) level were normal. The patient denied any recent antibiotic use or alcohol intake. He was admitted as a gastroenterology outpatient four days later. Upon examination, there were no abnormal clinical signs. The serum biochemical index was reexamined, GGT level was also markedly elevated (402 U/L), ALP level was mildly increased (162 IU/L), and the levels of ALT, AST, CPK, TBIL, direct bilirubin (DBIL), and indirect bilirubin (IBIL) were normal. Abdominal color Doppler ultrasound and computed tomography were obtained, and the results showed hepatic cysts. The serum alpha-fetoprotein (AFP) level was also normal, and tests for viral markers associated with hepatitis A virus, hepatitis B virus, hepatitis C virus, hepatitis E virus, anti-nuclear antibodies (ANAs), and anti-smooth muscle antibody (ASMA) were all negative. His abnormal hepatic function was diagnosed as atorvastatin-induced hepatic injury. Atorvastatin therapy was discontinued, and no other drugs were used to treat abnormal hepatic function. On day 21, the patient returned to the hospital and underwent blood tests. Results revealed serum GGT levels were significantly decreased (152 U/L), and the levels of ALT and ALP were normal. The GGT level was normal 3 weeks later (Fig. 1 GGT levels during atorvastatin therapy).

Causality assessment in adverse drug reactions is challenging in clinical settings. The Naranjo Adverse Drug Reactions Probability Scale is a simple and widely used method of assessing the adverse drug reactions (ADR) in drug-induced hepatotoxicity.[^7^,^8^] According to the Naranjo scale, the probability of ADR is classified as definite (score over 9), probable (score 5–8), possible (score 1–4), or doubtful (0 or lower). With Naranjo's assessment scale, the whole process was entered into the ADR evaluation system, and eventually scored 6 points (Table 1). The probability of GGT elevation was classified as probable by atorvastatin.

**Table 1:**
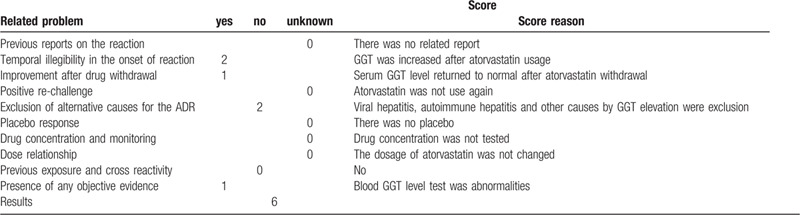
Naranjo's probability scale for assessing atorvastatin-associated GGT elevation.

## 3 Discussion

Muscle symptoms and hypertransaminasemia are common side effects of atorvastatin. There have been no reported that GGT elevation associated with atorvastatin in the absence of hyperbilirubinemia, hypertransaminasemiam, and CPK abnormalities. The liver function of this patient was normal when atorvastatin was initially used, and the level of GGT was significantly higher when atorvastatin was given for 4 weeks. The clinical pharmacist considered that the abnormally high GGT was associated with atorvastatin when viral hepatitis and autoimmune hepatitis were ruled out. This conclusion was also supported by Naranjo's assessment scale.

Up to 3% of patients develop a mild increase in transaminase within first year of statin therapy, but clinically apparent drug-induced liver injury is rare, and these increases often occur without symptoms and resolve spontaneously despite continued statin therapy.[^9^,^10^,^11^] The elevation in aminotransferases levels in the absence of elevated bilirubin is not clearly linked to clinically or pathologically relevant liver injury.^[12]^ In clinical practice, the biomarkers include ALT, TBL, and ALP, which are the standard analytes used to indicate liver damage and liver dysfunction in drug-induced liver injury.[^4^,^13^] According to European Association for the Study of the Liver clinical practice guidelines, there are three patterns of drug-induced liver injury: hepatocellular (5-fold or higher rise in ALT alone or when the ratio of serum activity of ALT to ALP is 5 or more), cholestatic (2-fold or higher rise in ALP alone or when the ratio of serum activity of ALT to ALP is 2 or lower), and mixed (the ratio of the serum activity of ALT to ALP is between 2 and 5). The ratio of ALT to ALP was less than 2 in this patient, but the levels of ALT and ALP were only slightly higher than normal upper limits, and the GGT level showed an abnormal increase. This raised the question of whether these changes could be explained by cholestasis induced by atorvastatin.

GGT is a microsomal enzyme located in the bile canaliculi, heart, kidney, lungs, pancreas, and seminal vesicles.^[14]^ The expression of GGT is essential to maintaining cysteine levels in the body. It has been reported that GGT plays an important role in the hepatobiliary system. The serum level of GGT has been used as an indicator of hepatic dysfunction and biliary tract disease, although the pathophysiological role of GGT itself remains unclear.^[15]^ The level of increase in GGT also often indicates acute hepatocyte damage. It is considered a preclinical and clinical biomarker for hepatotoxicity and hepatic injury.^[16]^ Here are some other effects of GGT elevation in clinical settings. A high serum level of GGT is associated with an increased risk of cancer in clinical trials, such as liver cancer and pancreatic cancer.[^17^,^18^] Enzymatic activity is also increased by enzyme-inducing drugs In the absence of other causes of liver disorder, such as antitubercular agents, antitumor drugs, and herbal remedies, enzymatic activity can be increased by enzyme-inducing drugs.[^19^,^20^] Basic experiments have shown the serum concentration of GGT can also act as a marker of oxidative stress, the extent of endothelial injury, and endothelial repair activity.^[21]^ The clinical usability of serum GGT as a marker of the development of cardiovascular disease is here explored. Shimizu et al. found that GGT had an association with hypertension and atherosclerosis.^[22]^ Abdominal imaging did not show cancer, and coronary CT angiography showed only slight atherosclerosis in this patient. The serum GGT level decreased to normal when atorvastatin was terminated. Therefore, we considered the abnormally high GGT level associated with atorvastatin.

Atorvastatin is largely metabolized in the liver via CYP 3A4. Genetic polymorphisms in CYP 3A4 may reflect differences in drug reactions. Dujovne et al. found that atorvastatin had more pronounced activity in lowering serum lipoprotein through CYP 3A4. this, in turn, could influence the structure of cellular membranes, leading to greater leakage of cellular enzymes (such as ALT, AST, ALP, and GGT) and increased incidence of liver-function test abnormalities without direct hepatotoxicity.^[23]^ Pitavastatin is minimally metabolized through CYP 450 enzymes,^[24]^ this might be the reason why the liver-function test was normal in this patient after given pitavastatin 2 months.

## 4 Conclusion

Although the side effects of atorvastatin have been described thoroughly in the literature, atorvastatin continues to appear to cause GGT elevation, especially in the absence of hyperbilirubinemia, hypertransaminasemia, and CPK abnormalities. Hence, clinicians should be aware of these possible adverse effects and monitor liver function tests in patients on statin therapy.

## Author contributions

**Conceptualization:** Yan Xu.

**Supervision:** Yanqing Wu.

**Writing – original draft:** Yan Xu.

**Writing – review editing:** Yanqing Wu.
